# Isorhapontigenin Inhibits Cell Growth, Angiogenesis, Migration, and Invasion of Non-Small-Cell Lung Cancer Cells Through NEDD9 Signaling

**DOI:** 10.3390/ijms26094207

**Published:** 2025-04-29

**Authors:** Zhuo Zhang, Jingxia Li, Daneah Willis, Sophia Shi, Huailu Tu, Max Costa

**Affiliations:** Division of Environmental Medicine, Department of Medicine, New York University Grossman School of Medicine, 341 E 25th Street, New York, NY 10010, USA

**Keywords:** lung cancer, dietary compound, angiogenesis, cancer cell growth, metastasis

## Abstract

Lung cancer is the leading cause of cancer deaths among American men, even though various treatments are available. The discovery and use of new alternative drugs to treat lung cancers are needed to reduce lung cancer mortality. Phytochemicals are potentially desirable therapeutic agents due to their better safety profiles. Isorhapontigenin (ISO) is an orally bioavailable dietary stilbene. Our studies show that treatment with ISO inhibits human lung cancer cell growth, angiogenesis, invasion, and migration. Neural precursor cell expressed developmentally downregulated 9 (NEDD9), a multi-domain scaffolding protein, regulates various processes crucial for tumorigenesis and metastasis. Our results show that NEDD9 is upregulated in the lung tissues from human lung adenocarcinomas (LUADs) and squamous-cell carcinomas (LUSCs) compared to normal lungs. Overexpression of NEDD9 elevates the invasion and migration of human lung cancer cells. Treatment of human lung cancer cells with ISO decreases NEDD9 protein levels. Our studies have also demonstrated that NEDD9 positively regulates angiogenesis, an essential factor in cancer progression. ISO treatment reduces angiogenesis. Moreover, ISO reduces the protein levels of hypoxia-inducible factor-1α (HIF-1α), a transcription factor critical for angiogenesis. Aberrant high expression of β-Catenin leads to various diseases including cancer. Our results show that ISO treatment reduces the activation of β-Catenin through the downregulation of NEDD9. Studies indicate that ISO decreases NEDD9, causing the suppression of cell growth, angiogenesis, invasion, and migration of human lung cancer cells. ISO is a potent therapeutic agent for lung cancer treatment.

## 1. Introduction

Lung cancer is the second most common cancer in both men and women in the United States [1]. It is classified into two main types, non-small-cell lung cancer (NSCLC) and small-cell lung cancer (SCLC) [2]. NSCLC, which accounts for about 80% to 85% of all lung cancers, is further classified into three major subtypes based on histological characteristics: adenocarcinoma, squamous-cell carcinoma, and large-cell carcinoma [2]. In 2020, 603,989 individuals were diagnosed with lung cancer in the U.S. [3]. Despite the availability of various treatment options, including chemotherapy, targeted therapy, and immunotherapy, lung cancer has remained the leading cause of cancer deaths among American men since the early 1950s and surpassed breast cancer as the leading cause of cancer deaths among American women in 1987 [4]. Therefore, the discovery and implementation of new alternative therapeutic agents are urgently needed to reduce lung cancer mortality. Phytochemicals are potentially desirable therapeutic agents because of their better safety profiles. Despite the promising effects of the phytochemical resveratrol against various cancers in vitro and in pre-clinical models, its poor bioavailability has limited its use and efficacy [5]. Thus, generating resveratrol derivatives with greater bioavailability as therapeutic targets is promising. Isorhapontigenin (ISO) is an orally bioavailable dietary stilbene among the derivatives. ISO is found in diverse natural resources, such as blueberries, grapes, and red wines [6]. A single oral administration of ISO at 100 µmol/kg is rapidly absorbed and has desirable marked persistence in the systemic circulation [7]. Daily dosing of ISO for one week has demonstrated its pharmacokinetic profile superiority to resveratrol [7]. ISO has been reported to exhibit multiple anticancer activities, including anti-inflammation [8], the ability to induce cell cycle arrest [9], promoting apoptosis [10], and suppressing the invasion of human cancer cells [11].

Neural precursor cell expressed developmentally downregulated 9 (NEDD9), also known as HEF1, is a multi-domain scaffolding protein that organizes signaling complexes involved in key processes of tumorigenesis and metastasis [12,13]. In cancer, NEDD9 overexpression enhances cell migration by promoting focal adhesion turnover; activates proliferative signal pathways; and contributes to genomic instability [14]. Due to its multiple roles in cancer progression, NEDD9 has been proposed as a potential therapeutic target. However, targeting NEDD9 poses a challenge, as it is located intracellularly and lacks kinase or catalytic domains. Thus, identifying dietary compounds capable of downregulating NEDD9 is of therapeutic interest.

β-Catenin, a versatile protein, is crucial in preserving physiological homeostasis. Abnormal regulation of β-Catenin has been associated with numerous diseases, especially cancer [15]. Hyperactivation of the Wnt/β-Catenin pathway drives uncontrolled cell proliferation, contributing significantly to tumorigenesis and impacting response to therapy [16]. Within the canonical Wnt signaling pathway, β-Catenin, acting as a central mediator, regulates Wnt-responsive genes by delivering signals to the nucleus [17]. Moreover, β-Catenin also serves as a scaffolding protein, facilitating interactions with multiple binding partners [18]. NEDD9 is involved in the canonical Wnt/β-Catenin pathway, controlling colon cancer cell progression [19].

Tumors rely on angiogenesis for both growth and metastasis. In the absence of a blood supply, cancer cells only grow 1–2 mm^3^ in size, but they can expand beyond 2 mm^3^ where angiogenesis occurs [20]. Without vascular support, tumors undergo necrosis or apoptosis [21]. As a result, angiogenesis is a critical factor in cancer progression. Vascular endothelial growth factor (VEGF) is a primary driver of this process. Under hypoxic conditions, VEGF and its receptor were upregulated through hypoxia-inducible factor-1α (HIF-1α) [22]. HIF-1α plays a vital role in promoting tumor survival and advancement [23], and its levels are elevated in over half of primary human cancers and their metastases [24]. Research indicates that NEDD9 upregulates HIF-1α, promoting hypoxia-induced migration of colorectal cancer cells [25].

The present study examined how ISO inhibits cell growth/survival, angiogenesis, invasion, and migration via NEDD9 signaling.

## 2. Results

### 2.1. ISO Inhibited Cell Growth/Survival, Angiogenesis, Migration, and Invasion

**Figure 1:**
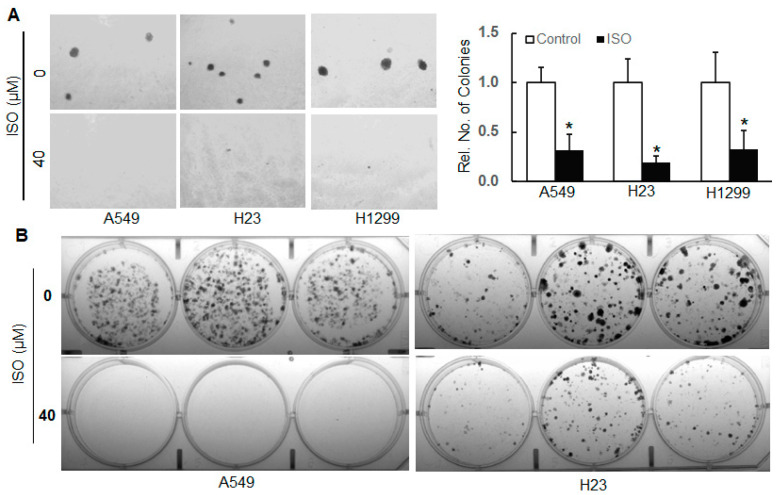
ISO inhibited the cell growth/survival of human lung cancer cells. (**A**) Human lung cancer cell lines A549, H23, and H1299 were subjected to soft agar assay in the presence and absence of ISO treatment (40 µM). After about 4 weeks, images were captured by an Olympus microscope, and numbers of colonies were recorded. *, *p* < 0.05, compared to those without ISO treatment. (**B**) A549 and H23 cells with and without ISO treatment (40 µM) were subjected to a clonogenicity assay. After about 2 weeks, images were captured by a Molecular Imager.

Disruption of the mammalian cell cycle contributes to cellular transformation. Our results from an anchorage-independent growth assay show that ISO treatment markedly reduced the number of colonies in A549, H23, and H1299 cells (Figure 1A). In addition, a clonogenicity assay was used to evaluate the cell survival of these lung cancer cell lines following ISO treatment. The results show that ISO treatment dramatically reduced cell survival in A549 and H23 cells (Figure 1B). Our previous studies indicated that treating human bronchial epithelial BEAS-2B cells with ISO at 40 µM up to 72 h did not cause any measurable cytotoxicity [26].

**Figure 2:**
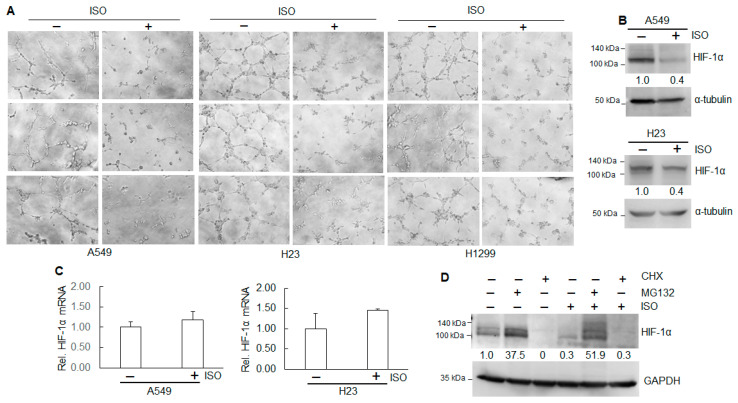
ISO inhibited the angiogenesis of human lung cancer cells. (**A**) Human lung cancer cell lines A549, H23, and H1299 with or without ISO treatment (40 µM) were subjected to an in vitro tube formation assay. Images were captured using an Olympus microscope. (**B**,**C**) A549 and H23 cells were treated with 40 µM ISO for 24 h. The whole-cell lysates were collected for immunoblotting analysis (**B**). RNAs were isolated for qRT-PCR (**C**). (**D**) A549 cells were treated with or without 40 µM ISO in combination with 20 µM MG132 or 50 µg/mL CHX. The whole-cell lysates were collected for immunoblotting analysis. (**B**,**D**) The band intensity was evaluated using ImageJ (version 1.53m). The data represent the relative intensity after normalization.

As solid tumors grow greater than 1–2 mm^3^ in size, they create a microenvironment characterized by hypoxia, ischemia, and increased interstitial pressure. In response, tumor tissues secrete growth factors that trigger angiogenesis to sustain continued growth [27,28,29]. Angiogenesis, the formation of new blood vessels, regulated by multiple pro-angiogenic factors, is crucial for tumor growth, as well as for promoting metastasis [30]. A tube formation assay was carried out to examine ISO’s effect on in vitro angiogenesis. The results show that ISO treatment markedly reduced the numbers and length of tubes in A549, H23, and H1299 cells (Figure 2A), indicating that ISO can inhibit angiogenesis. Hypoxia is a common feature of most tumors, which contributes to epithelial–mesenchymal transition (EMT), drug resistance, and tumor progression through HIF-1α [31]. Our results show that ISO treatment decreased the HIF-1α protein levels in A549 and H23 cells (Figure 2B). However, ISO did not alter the HIF-1α mRNA levels (Figure 2C). Further study indicates that MG132, a proteasome inhibitor that prevents protein degradation, elevated the HIF-1α protein levels in A549 cells treated with or without ISO (Figure 2D). Cycloheximide (CHX), which inhibits protein synthesis, completely abolished HIF-1α expression (Figure 2D). Those results indicate that ISO downregulates HIF-1α protein through translation impairment and post-translational modification.

**Figure 3:**
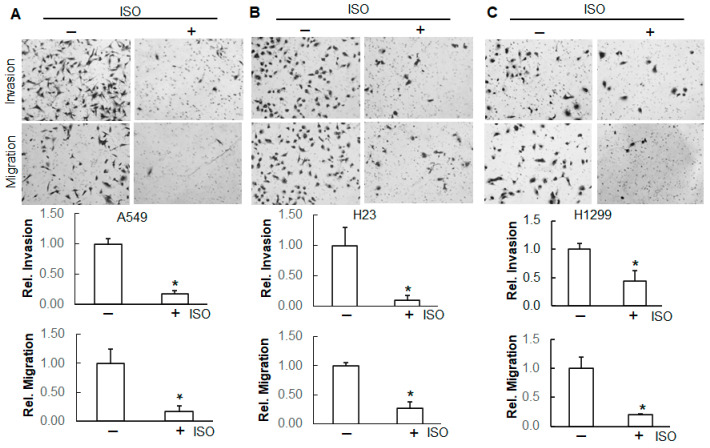
ISO inhibited the invasion and migration of human lung cancer cells. A549 (**A**), H23 (**B**), and H1299 cells (**C**) with and without ISO treatment (40 µM) were subjected to transwell invasion and migration assay. After 48 h, the images were captured using an Olympus microscope. Invaded and migrated cells were counted and recorded. *, *p* < 0.05, compared to those without ISO treatment.

Metastasis is a hallmark of cancer. A transwell assay was used to examine the effect of ISO on the invasion and migration of these cancer lines. Our results show that ISO treatment significantly decreased the number of invaded and migrated cells (Figure 3), indicating that ISO suppresses the invasion and migration of these lung cancer cells.

The above data demonstrate that ISO can reduce the malignancy of lung cancer cells, including A549, H23, and H1299 cells.

### 2.2. Elevated Expression of NEDD9 in Human Lung Tumor Tissues

**Figure 4:**
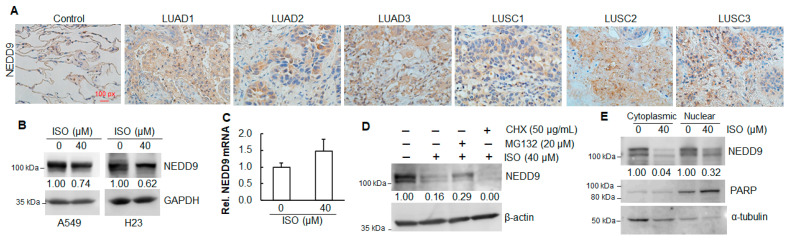
NEDD9 was upregulated in human lung cancer tissues, and ISO decreased NEDD9 levels in human lung cancer cells. (**A**) Formalin-fixed human lung tissues were subjected to immunohistochemistry staining. (**B**) A549 and H23 cancer cells were treated with 40 µM ISO for 24 h. The whole-cell lysates were collected for immunoblotting analysis. (**C**) A549 cells were treated with 40 µM ISO for 24 h. RNAs were isolated and subjected to qRT-PCR. (**D**) A549 cells were treated with or without 40 µM ISO in combination with MG132 or CHX. The whole-cell lysates were collected for immunoblotting analysis. (**E**) A549 cells were treated with 40 µM ISO for 24 h. The cytoplasmic and nuclear proteins were isolated and subjected to immunoblotting analysis. (**B**,**D**,**E**) The band intensity was quantified using ImageJ (version 1.53m), followed by normalization.

NEDD9 is a multi-domain scaffold protein that coordinates signaling complexes responsible for regulating cell survival, adhesion, and migration [32,33]. To measure the expression levels of NEDD9 in human primary lung tumor tissues as well as normal tissues, an immunohistochemistry assay was conducted in the tissues from human lung adenocarcinoma (LUAD) and squamous-cell carcinoma (LUSC) with Grades 1, 2, and 3. The results show that the NEDD9 levels were increased in the tissues of LUAD and LUSC compared to those in normal lung tissues (Figure 4A). The expression levels of NEDD9 remained similar among the tissues from Grades 1, 2, and 3 in both LUAD and LUSC (Figure 4A).

### 2.3. ISO Decreased NEDD9 Protein Levels in Human Lung Cancer Cell Lines

NEDD9 has been reported to enhance the invasion and migration of cervical carcinoma cells [34]. Our results show that ISO suppressed invasion and migration in human lung cancer cells (Figure 3A–C). To test whether ISO can downregulate NEDD9, the levels of NEDD9 protein and mRNA were measured. The results show that ISO treatment decreased the NEDD9 protein levels (Figure 4B), not mRNA levels (Figure 4C). Treatment with MG132 somehow restored the NEDD9 protein, which was reduced by ISO (Figure 4D). Treatment of the cells with ISO and CHX further decreased the NEDD9 protein levels (Figure 4D). The above data suggest that the downregulation of NEDD9 by ISO is through translation and post-translational modification. The nucleus functions as the cell’s control center and houses its genetic material. We have examined the NEDD9 levels in both the cytoplasm and nucleus. The results show that NEDD9 was expressed in the cytoplasm and nucleus (Figure 4E). ISO treatment reduced the NEDD9 levels in the cytoplasm and nucleus (Figure 4E).

### 2.4. Inhibition of ISO on Cell Growth and Angiogenesis Is Through NEDD9

**Figure 5:**
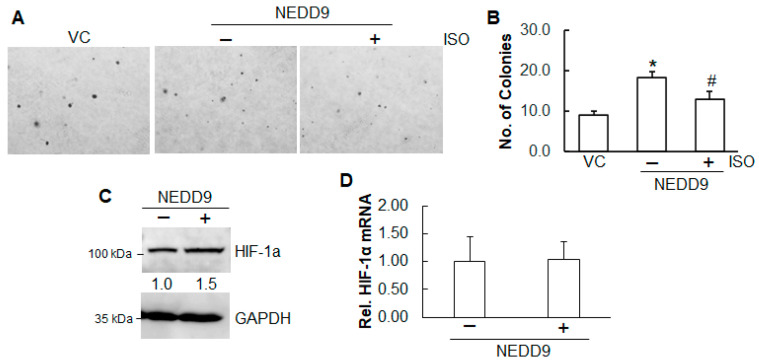
Inhibition of ISO on cell growth and angiogenesis is through NEDD9. (**A**,**B**) A549 cells with or without stable expression of NEDD9 in combination with ISO treatment (40 µM) were subjected to soft agar assay. The images were captured (**A**). Colonies were counted and recorded (**B**). * and #, *p* < 0.05, compared to the cells without stable expression of NEDD9 and the cells with stable expression of NEDD9 but without ISO treatment, respectively. (**C**) The whole-cell lysates in A549 cells with or without stable expression of NEDD9 were collected for immunoblotting analysis. The band intensity was evaluated using ImageJ (version 1.53m), followed by normalization. (**D**) RNAs were isolated from A549 cells with or without stable expression of NEDD9. qRT-PCR was conducted to measure HIF-1α mRNA levels.

Our results show that ISO inhibited anchorage-independent growth (Figure 1) and angiogenesis (Figure 2) and that ISO reduced NEDD9 expression (Figure 4B). To test whether the downregulation of NEDD9 plays a role in cell growth and angiogenesis, stable overexpression of NEDD9 in A549 cells was established. The results from the anchorage-independent growth assay show that overexpression of NEDD9 promoted colony formation (Figure 5A,B). More importantly, upon ISO treatment, the cells with overexpression of NEDD9 grew more colonies than cells with vector control (Figure 5A,B).

Similarly, the role of NEDD9 in ISO-inhibited HIF-1α was also examined. The results show that in A549 cells, overexpression of NEDD9 elevated the HIF-1α protein levels (Figure 5C), not its mRNA levels (Figure 5D), suggesting that NEDD9 is a positive regulator of HIF-1α.

### 2.5. ISO Suppressed Migration and Invasion Through NEDD9/β-Catenin Signaling

**Figure 6:**
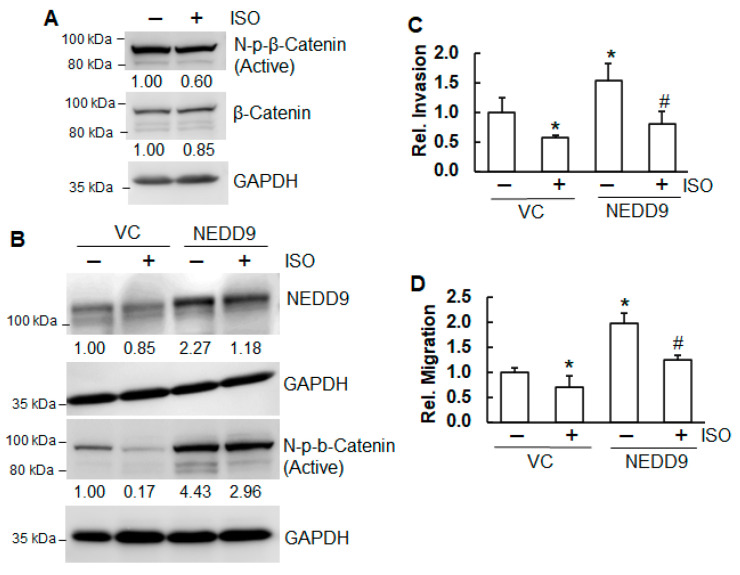
ISO suppressed migration and invasion through NEDD9/β-Catenin signaling. (**A**) A549 cells were treated with 40 µM ISO for 24 h. The whole-cell lysates were collected for immunoblotting analysis. (**B**) A549 cells with and without stable expression of NEDD9 treated with 40 µM ISO for 24 h were subjected to immunoblotting analysis. The band intensity was evaluated using ImageJ (version 1.53m), followed by normalization. (**C**,**D**) A549 cells with and without stable expression of NEDD9 in combination with ISO treatment (40 µM) were subjected to a transwell invasion and migration assay. After 48 h, cells were fixed and stained. Images were captured using an Olympus microscope. Invaded (**C**) and migrated cells (**D**) were counted and recorded. * and #, *p* < 0.05, compared to the cells without stable expression of NEDD9 and the cells with stable expression of NEDD9 but without ISO treatment, respectively.

The Wnt/β-Catenin signaling pathway governs cancer cell proliferation, migration, and invasion [35]. Our results show that treatment of A549 cells with ISO decreased non-p-β-Catenin (N-p-β-Catenin), an active form of β-Catenin (Figure 6A). Our previous studies have demonstrated that NEDD9 positively regulates N-p-β-Catenin, suppressing the invasion and migration of A549 cells [36]. NEDD9 overexpression blocked the reduction in N-p-β-Catenin by ISO (Figure 6B). Moreover, NEDD9 overexpression promoted invasion and migration (Figure 6C,D). The inhibitory effect of ISO on invasion and migration was markedly reduced by NEDD9 overexpression (Figure 6C,D). The above results suggested that NEDD9 positively regulated β-Catenin and that, through a decrease in NEDD9, ISO inhibited the activation of β-Catenin, thus suppressing invasion and migration.

c-Myc, a proto-oncogene, has been recognized as a downstream target of the Wnt/β-Catenin pathway [37]. It has been reported that activation of the canonical Wnt signaling pathway stimulates cyclin D1 gene transcription, thus facilitating the G1/S phase transition and cell cycle progression [38]. Our results show that ISO treatment in A549 cells decreased the expression of both c-Myc and Cyclin D1 (Supplemental Figure S1). Overexpression of NEDD9 increased c-Myc and Cyclin D1 expression; however, overexpression of NEDD9 failed to restore c-Myc and Cyclin D1 inhibited by ISO (Supplemental Figure S1). Those data indicate that NEDD9 positively regulates c-Myc and Cyclin D1, and the downregulation of c-Myc and Cyclin D1 by ISO is independent of NEDD9.

## 3. Discussion

While surgery remains the most curative treatment option for patients with NSCLC, postoperative chemotherapy offers additional survival benefits. Over the past few decades, significant advancements have been made in NSCLC management, including introducing targeted therapies for oncogene-driven NSCLC and immune checkpoint inhibitors (ICIs) [2]. Identifying pathogenic variants has been instrumental in developing molecularly targeted therapies, which have improved survival outcomes in select patients with metastatic disease. Notably, genetic alterations in the EGFR, MAPK, and PI3K signaling pathways can impact drug sensitivity and contribute to primary and acquired resistance to kinase inhibitors. For patients with advanced-stage disease, chemotherapy or EGFR kinase inhibitors provide only modest survival benefits [39]. Additionally, radiotherapy has expanded the role of local ablative treatments, especially for patients with oligometastatic disease. Despite progress, the development of acquired resistance to targeted therapies remains a major challenge. Nonetheless, emerging drug strategies aimed at overcoming resistance are helping to expand the range of available treatment options.

Studies have highlighted the potential of resveratrol in combating various cancers; however, its limited bioavailability has hindered its clinical utility and effectiveness [5]. Consequently, the development of resveratrol derivatives with greater bioavailability but comparable biological activity is of great interest. Isorhapontigenin (ISO) (trans-3,5,4′-trihydroxy-3′methoxystilbene), a methoxylated resveratrol derivative, stands out as an orally bioavailable dietary stilbene with promising anticancer potential [7]. Beyond its anticancer properties, ISO has also been demonstrated to have anti-inflammation [40], antiviral [41], cardioprotective [42], neuroprotective [43], and antidiabetic properties [44].

ISO exhibits a broad range of anticancer effects through various mechanisms. In cancer cells, it induces G0/G1 phase arrest and promotes apoptosis [9,10]. Specifically, ISO suppresses cyclin D1 expression, thereby hindering cell cycle progression in prostate cancer cells [10]. In human bladder cancer cells, ISO has been shown to reduce the levels of the anti-apoptotic protein XIAP, leading to enhanced apoptosis [45]. Furthermore, ISO increases the expression of pro-apoptotic proteins such as Bax and cleaved caspase-3, further promoting apoptosis [45]. The present study indicates that ISO downregulates c-Myc and Cyclin D1 and suppresses the anchorage-independent growth of human lung cancer cells.

NEDD9 has been identified as a biomarker of aggressive human tumors, including melanoma [46], breast [47], and lung [48] cancers. An immunostaining analysis of 60 formalin-fixed and paraffin-embedded lung adenocarcinoma tissues revealed a statistically significant difference in NEDD9 expression levels between metastatic and non-metastatic lung adenocarcinomas [48]. The results from a fluorescent quantitative reverse transcription polymerase chain reaction (fq-PCR) indicated a 10- to 100-fold increase in NEDD9 mRNA expression in highly invasive lung adenocarcinoma cell lines compared to the less invasive cell lines [48]. We examined the NEDD9 expression levels from the tissues of human lung adenocarcinomas (LUADs) and squamous-cell carcinomas (LUSCs), Grades 1–3. Not surprisingly, the expression levels of NEDD9 in the tissues from LUADs and LUSCs were all elevated compared to the normal lung tissues. However, among the tumor tissues from Grades 1, 2, and 3 of both LUADs and LUSCs, the NEDD9 levels remained similar.

NEDD9 plays a vital role in several oncogenic pathways. As a scaffold protein, it facilitates the activation of key signaling molecules, including Src, one of the first discovered oncogenes. Silencing NEDD9 has been shown to reduce STAT3 activation, thereby suppressing ovarian tumor growth [49]. Tumorigenesis is often associated with the Warburg effect, where cancer cells rely heavily on glycolysis for ATP production rather than oxidative phosphorylation. One study demonstrated that NEDD9 depletion disrupts glycolysis by impairing the expression and activation of several glycolytic enzymes, both in vitro and in vivo [50]. Our findings show that overexpression of NEDD9 increased anchorage-independent growth, and the inhibitory effect of ISO was reduced by NEDD9 overexpression. Collectively, these findings suggest that NEDD9 contributes to multiple oncogenic processes, and ISO may inhibit anchorage-independent growth, at least in part, by targeting NEDD9.

Cancer cells initiate angiogenesis by signaling surrounding tissues and activating growth factors that stimulate the formation of new blood vessels, thus supporting the rapid growth of tumors. HIF-1α plays a crucial role in tumor survival and progression [23] and is elevated in over half of primary human cancers and their metastases [24]. HIF-1 is involved in all stages of angiogenesis, facilitating and sustaining the formation of new blood vessels throughout the process. Our results show that ISO decreased the HIF-1α protein levels. Overexpression of NEDD9 elevated the HIF-1α protein levels in A549 cells. Neither ISO treatment nor NEDD9 overexpression altered the HIF-1α mRNA levels, indicating that the downregulation of HIF-1α by ISO or its upregulation by NEDD9 is through translation and post-translational modification. Under normal oxygen conditions (normoxia), prolyl hydroxylase domain proteins (PHDs), which require oxygen for their function, hydroxylate HIF-1α proline residues through direct binding to its oxygen-dependent degradation (ODD) domain. The binding of hydroxylated HIF-1α to Von Hippel-Lindau (pVHL) causes ubiquitous degradation of HIF-1α [51]. We hypothesize that NEDD9 binds to the ODD domain of HIF-1α in competition with PHD2, resulting in reduced binding between PHD2 and HIF-1α and subsequent stabilization of HIF-1α, and that ISO reduces the binding and subsequent reduction in stabilization of HIF-1α. However, the precise mechanism needs to be further investigated.

β-Catenin is a highly conserved protein with key roles in both the canonical Wnt pathway and cell adhesion. In the Wnt/β-Catenin pathway, β-Catenin translocates to the nucleus, where it activates target genes through interaction with TCF/LEF transcription factors [15]. Aberrant accumulation of β-Catenin is a well-recognized contributor to the development of several cancers, notably colon and liver [52]. Regulating β-Catenin homeostasis has emerged as a promising approach for targeted cancer therapy [53]. The non-phosphor-β-Catenin (Ser45) (N-p-β-Catenin), an active form, represents a stabilized β-Catenin protein. Our results show that ISO treatment decreased N-p-β-Catenin. Overexpression of NEDD9 increased N-p-β-Catenin, indicating that NEDD9 positively regulates β-Catenin. Moreover, the inhibition of N-p-β-Catenin by ISO was reduced by NEDD9 overexpression, suggesting that the inhibition of N-p-β-Catenin by ISO is through NEDD9. Since glycogen synthase kinase-3 beta (GSK-3β) is a major regulator of β-Catenin, it is also possible that ISO increases GSK-3β activities, resulting in less stabilization of β-Catenin, which may contribute to reduced cell growth and proliferation.

Our previous study observed that ISO downregulated vimentin, one of the EMT markers, suppressing invasion in human bladder cancer cells [11]. The present study shows that ISO suppressed invasion and migration in human lung cancer cells. While the knockdown of NEDD9 reduced invasion and migration, overexpression of NEDD9 increased invasion and migration. Furthermore, the invasion and migration inhibited by ISO were restored by NEDD9 overexpression. Our data indicated that NEDD9 mediated the invasion and migration suppressed by ISO treatment. NEDD9 has been linked to the EMT across various tumor types [54,55]. It regulates key EMT markers, including the downregulation of E-Cadherin and the upregulation of N-Cadherin [54]. In human mammary cells, NEDD9 facilitates the removal of E-Cadherin from the cell membrane and its degradation via the lysosomes [55]. It is speculated that ISO downregulates NEDD9, causing upregulation of E-Cadherin, thus suppressing invasion and migration of lung cancer cells.

In summary, the present study has demonstrated that ISO can suppress the anchorage-independent growth, angiogenesis, invasion, and migration of human lung cancer cells. ISO decreases the NEDD9 protein levels, causing the downregulation of HIF-1α and reduction in β-Catenin activation, leading to the inhibition of the malignancy of human lung cancer cells. NEDD9 can be a potent therapeutic target for cancer treatment, and ISO is a candidate to inhibit NEDD9. 

## 4. Materials and Methods

### 4.1. Reagents

Dulbecco’s Modified Eagle Medium (DMEM) and RPMI 1640 Medium were purchased from Fisher Sci (Waltham, MA, USA). Basal medium Eagle was from Sigma (St Louis, MO, USA).

Transfection reagent PolyJet was purchased from SignaGen Laboratories (Rockville, MD, USA). Primary antibodies against NEDD9 (Cat# 4044), non-p-β-Catenin (Cat# 19807), β-Catenin (Cat# 9582), and secondary antibodies against mice (Cat# 7056) and rabbits (Cat# 7054) were purchased from Cell Signaling Inc. (Beverly, MA, USA). HIF-1α primary antibody (Cat# GTX131826) was purchased from GeneTex (Irvine, CA, USA). GAPDH (Cat# 60004-1-Ig) and α-tubulin (Cat# 66031-1-Ig) antibodies were purchased from Protein Technologies Inc. (Tucson, AZ, USA). All primary antibodies were diluted in 1:1000 using 5% milk or 5% BSA. A nuclear extraction kit and primary antibody against NEDD9 (Cat# ab18056) for immunohistochemistry staining were purchased from Abcam Biotech (Waltham, MA, USA). ECF substrate for Western blot was purchased from GE Healthcare (Pittsburgh, PA, USA).

### 4.2. Plasmids

NEDD9 shRNA (Addgene_21963 and Addgene_21964), NEDD9 overexpression (Addgene_21962), and their control vectors were purchased from Addgene (Cambridge, MA, USA).

### 4.3. Cell Culture and Stably Expressing Cells

Human lung adenocarcinoma epithelial cells A549 (Cat# CCL-185) and H23 (Cat# CRL-5800), and non-small-cell lung cancer large-cell carcinoma cells H1299 (Cat# CRL-5803) and human umbilical vein endothelial cells (HUVECs) (Cat# PCS-100-010) were purchased from ATCC (Manassas, VA, USA). A549 cells were grown in DMEM medium, and H23 and H1299 cells were grown in RPMI 1640 medium with 10% FBS. The cells were split when they reached 90% confluence. HUVEC cells were grown in an M199 medium supplemented with FBS and growth factors.

To establish stable expression or knockdown cells, the cells were transfected with 2 µg plasmid DNA in each well of a six-well plate, followed by antibiotic selection for at least one month. Gene expression was verified by immunoblotting analysis.

### 4.4. Immunoblotting Analysis

The cells were cultured in 6-well plates. After 90% confluence, the cells were lysed using a boiling buffer. The whole-cell lysates were sonicated. Protein concentrations were measured. Proteins were separated by SDS-PAGE gels, followed by overnight incubation with primary antibodies. The blots were probed with secondary antibodies. Proteins were visualized using an ECF substrate. The band intensity was evaluated using software ImageJ (version 1.53m).

### 4.5. Real-Time qPCR

RNA was extracted using Trizol reagent following the manufacturer’s instructions (Thermo Fisher Sci, Waltham, MA, USA). Briefly, incubate the homogenized samples for 5 min at room temperature, followed by adding 0.2 mL of chloroform per 1 mL of TRIZOL Reagent. Centrifuge the samples at 12,000× *g* for 15 min at 4 °C. Transfer the aqueous phase to a new tube. Precipitate the RNA from the aqueous phase by mixing with isopropyl alcohol. Wash RNA pellets once with 75% ethanol. Air-dry the RNA pellets. Dissolve RNAs using RNase-free water. cDNA for NEDD9 was synthesized using a SuperScript first-strand synthesis kit (Thermo Fisher Sci, Waltham, MA, USA). Primers were designed using Primer-Blast with forward sequence (F) and reverse (R) as illustrated: NEDD9: F-GATGGGTGTCTCCAGCCTAA, R-GGATCTGGTGGGAGTCTTCA; HIF-1α: F-TCCAAGAAGCCCTAACGTGT, R-TTTCGCTTTCTCTGAGCATTCTG; GAPDH: F- AGAAGGCTGGGGCTCATTTG, R-AGGGGCCATCCACAGTCTTC. NEDD9 mRNA levels were measured using PowerUp SYBR Green master mix (Thermo Fisher Sci, Waltham, MA, USA) and GAPDH as a control. The value of the cycle threshold (CT) was examined. Data were analyzed by calculation of ΔΔCT.

### 4.6. Soft Agar Assay

Soft agar assays were used to evaluate the anchorage-independent growth ability. Briefly, 3 mL of 0.5% agar in basal medium Eagle (BME) with 10% FBS was layered onto each well of 6-well tissue culture plates. An amount of 1 mL of 0.33% agar containing 2000–5000 cells (base agar) was added to each well on top of the 0.5% agar layer. Plates were incubated at 37 °C in 5% CO_2_. The images were captured, and the number of colonies was counted.

### 4.7. Clonogenicity Assay

Clonogenicity assay is used to examine in vitro cell survival based on the ability of a single cell to grow into a colony. Briefly, 2000 cells were seeded in the 6-well plates. Plates were incubated at 37 °C in 5% CO_2_. The cells were fixed with 3.7% formaldehyde, followed by staining with crystal violet (0.5% *w*/*v*). Images were captured using a Molecular Imager (Bio-Rad, Hercules, CA, USA).

### 4.8. Tube Formation Assay

Human umbilical vein endothelial cells (HUVECs) were grown in M199 medium with 15% FBS and growth factors. After 90% confluence, the cells were treated with condition medium harvested from A549, H23, and H1299 cells with or without ISO treatment (40 μM, 24 h). After 8 h, the images were captured. Three images from each group were selected as representatives.

### 4.9. Invasion and Migration Assays

The invasion transwell assay was conducted using the Biocoat Matrigel Invasion Chambers according to the manufacturer’s manual (Corning, Tewksbury, MA, USA). The migration assay was performed using chamber inserts (Corning, NY, USA). Amounts of 0.5–1.0 × 10^4^ cells per well were seeded in the chamber inserts in 500 μL of medium with 0.1% FBS. The inserts were placed into the wells with 700 µL culture medium supplemented with 10% FBS. After 48 h, the cells were washed and fixed with 3.7% formaldehyde and methanol, then stained with Giemsa. The images were captured using an Olympus microscope. The number of invaded and migrated cells was counted and recorded.

### 4.10. Nuclear Extraction

Cells were grown in 10 cm dishes. The cells were harvested after 90% confluence. The cell pellets were resuspended in a pre-extraction buffer. The cytoplasmic extract was collected. The nuclear pellets were resuspended in the extraction buffer. The nuclear supernatant was collected, followed by centrifugation at 14,000 rpm. The protein concentrations of cytoplasmic and nuclear fractions were measured.

### 4.11. Human Lung Tissues and Immunohistochemistry Staining

The Center for Biospecimen Research & Development at New York University Langone Health provided the human lung tissues. The normal lung tissues were from a 74-year-old female. The lung adenocarcinoma tissues (LUAD1-3) were from a 76-year-old male, a 64-year-old female, and a 78-year-old female, respectively. All three patients are smokers. The lung squamous carcinoma tissues (LUSC1-3) were from a 90-year-old male, a 70-year-old male, and a 78-year-old female, respectively. LUSC1 and LUSC3 are smokers, and LUSC2 has never smoked. The detailed procedure of immunohistochemistry staining was described previously [56]. The images were captured using a Nikon microscope. The intensity of the brown color represents the expression levels of the NEDD9 protein.

### 4.12. Statistical Analysis

The Student’s test was used to evaluate the difference between the two groups. A value of *p* < 0.05 was considered significant.

## Data Availability

The data generated in this study are available upon request.

## Supplementary Materials

The following supporting information can be downloaded at: https://www.mdpi.com/article/10.3390/ijms26094207/s1.
