# Supplementary figures and images for: Isorhapontigenin Inhibits Cell Growth, Angiogenesis, Migration, and Invasion of Non-Small-Cell Lung Cancer Cells Through NEDD9 Signaling
Source: Int J Mol Sci. 2025 Apr 29;26(9):4207. doi: 10.3390/ijms26094207 (PMC12071804; doi:10.3390/ijms26094207)

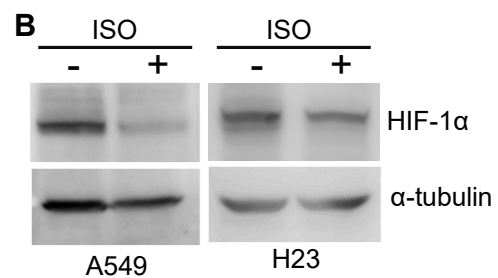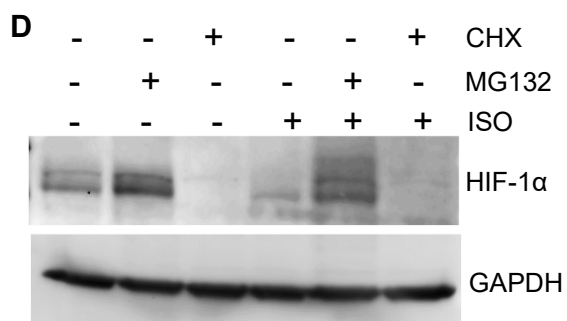

**Figure 2**

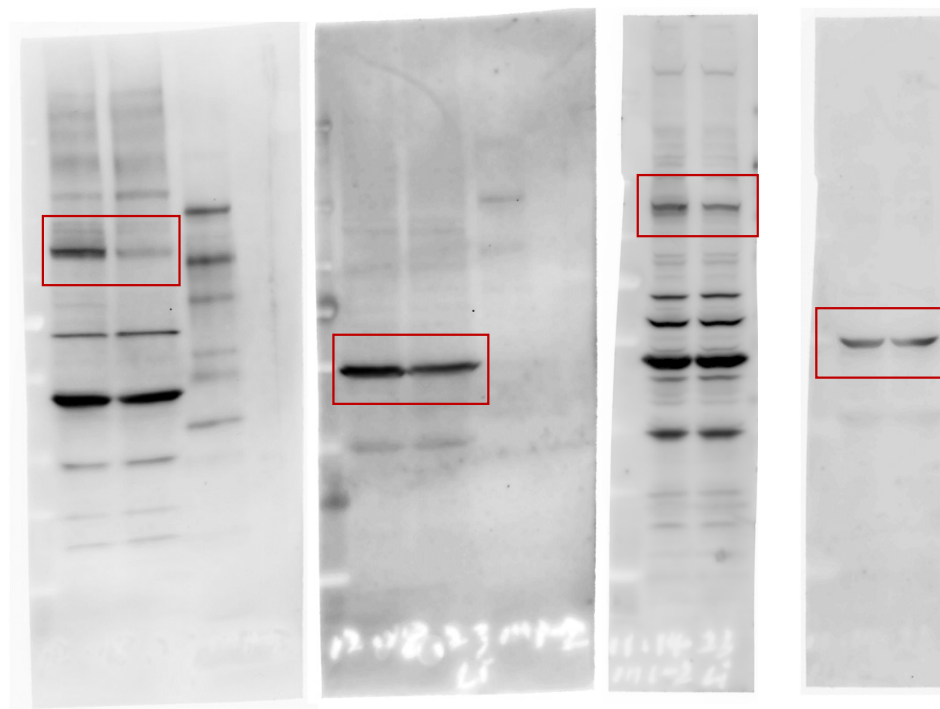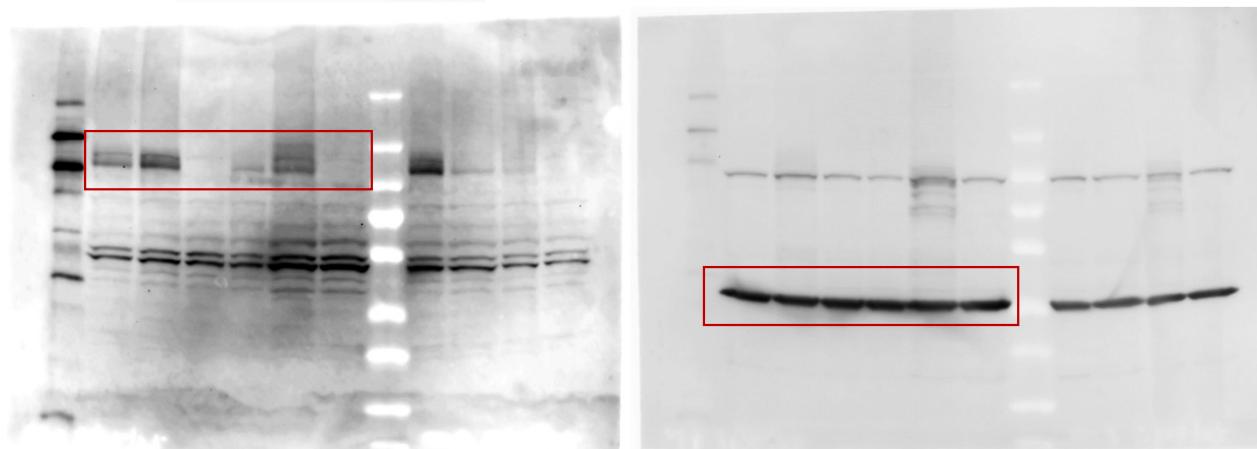

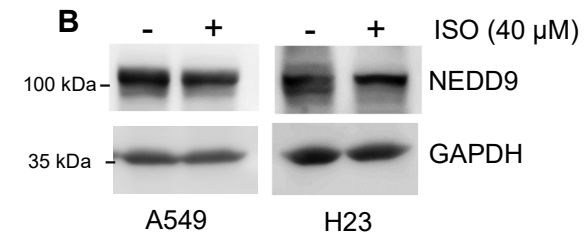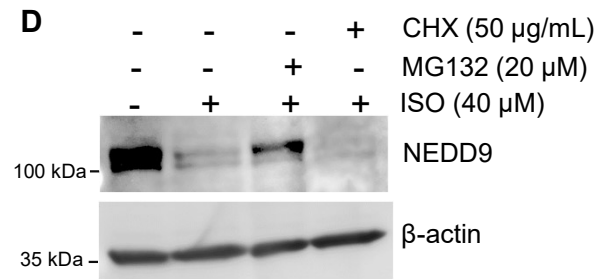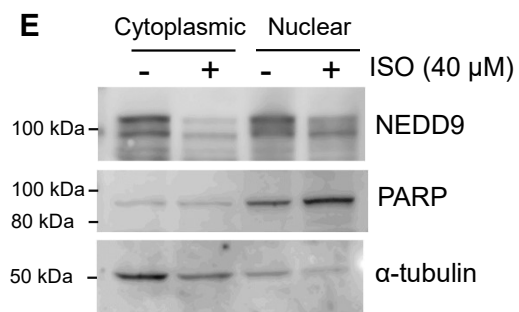

**Figure 4**

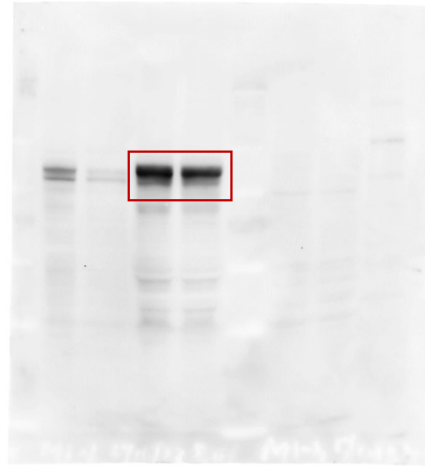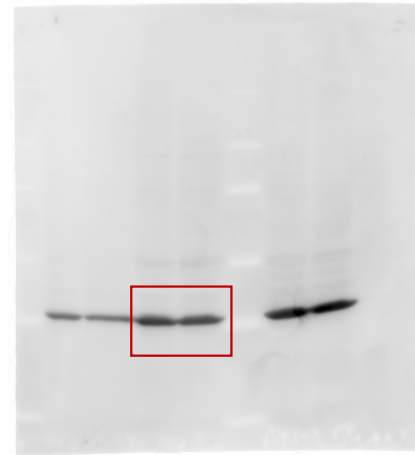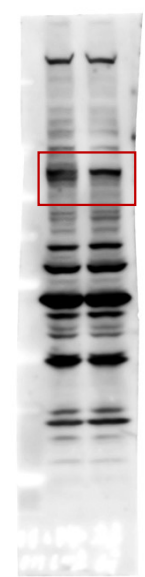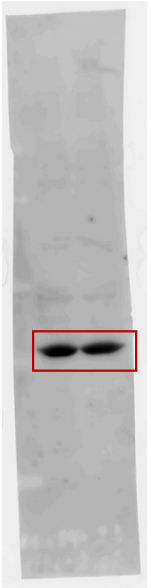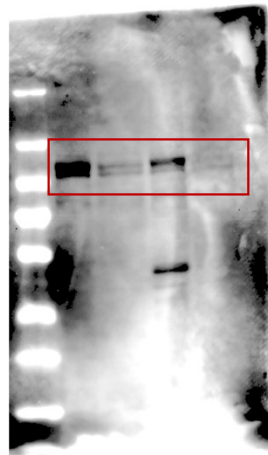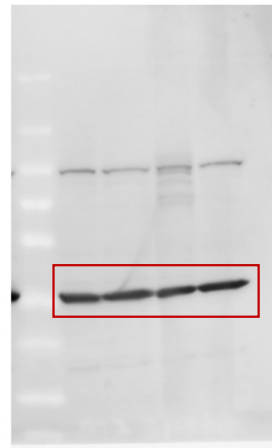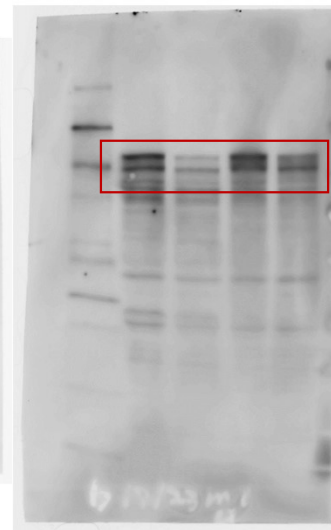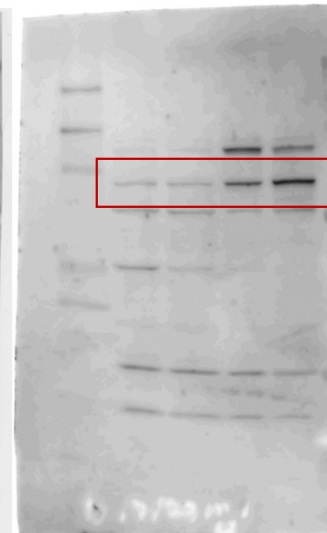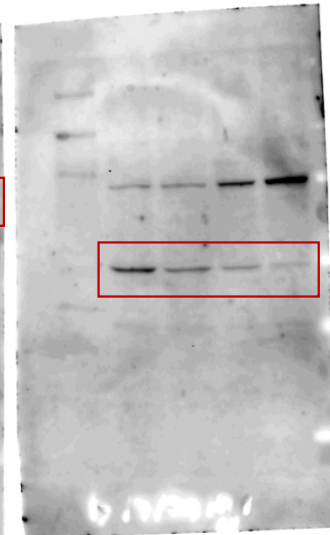

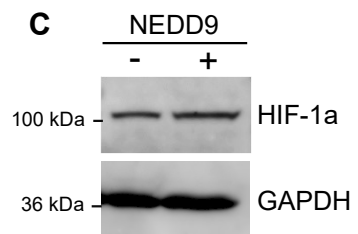

**Figure 5**

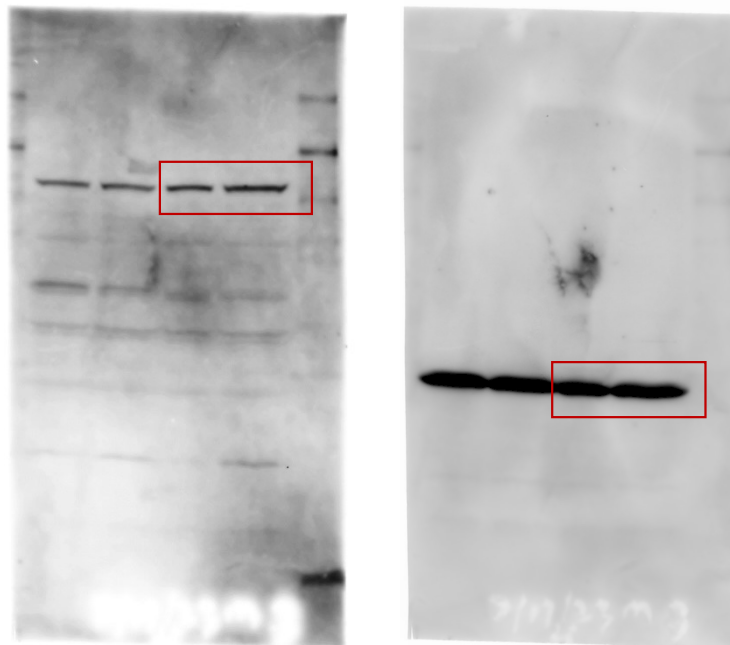

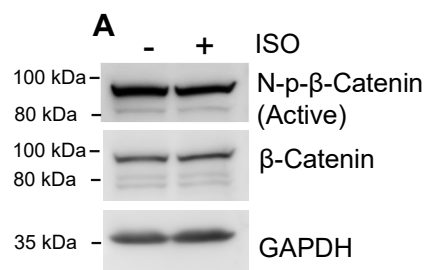

**Figure 6**

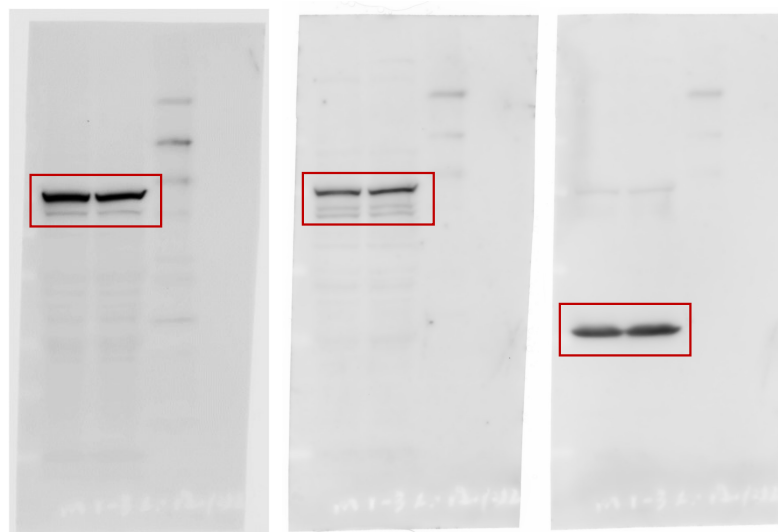

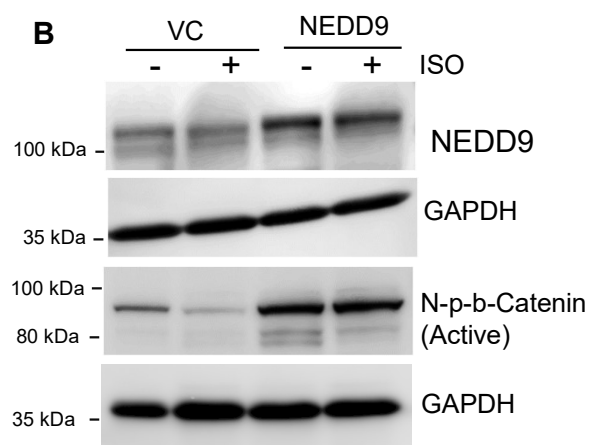

**Figure 6**

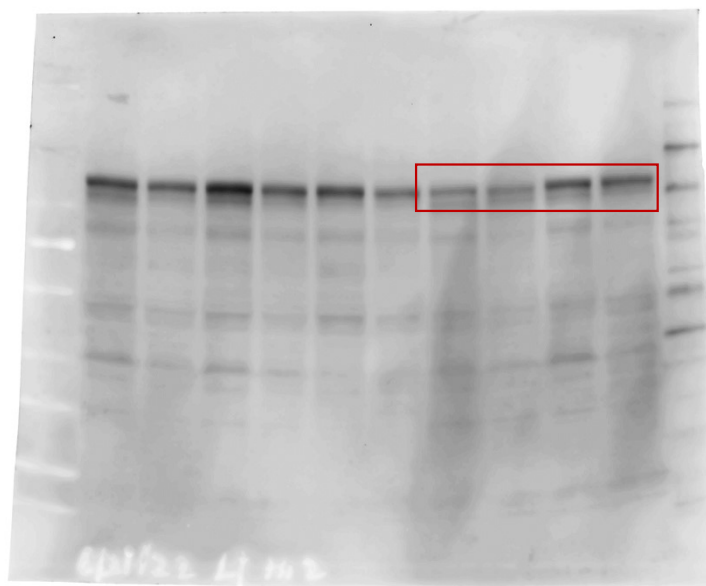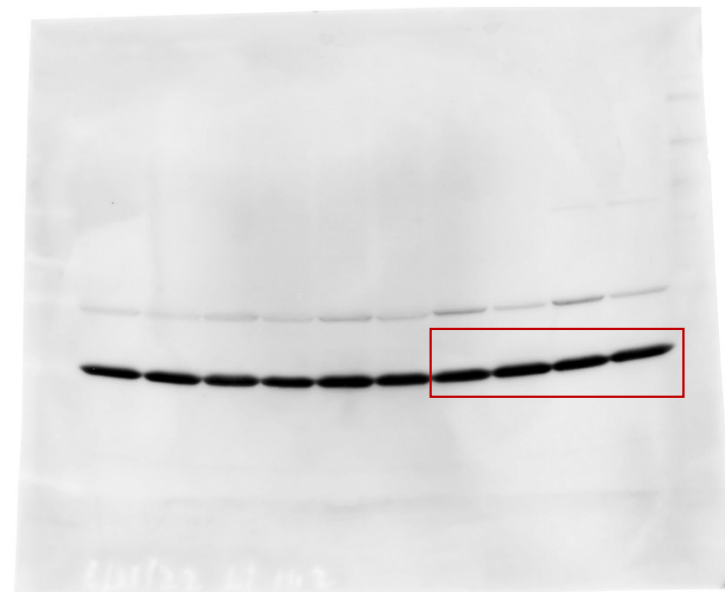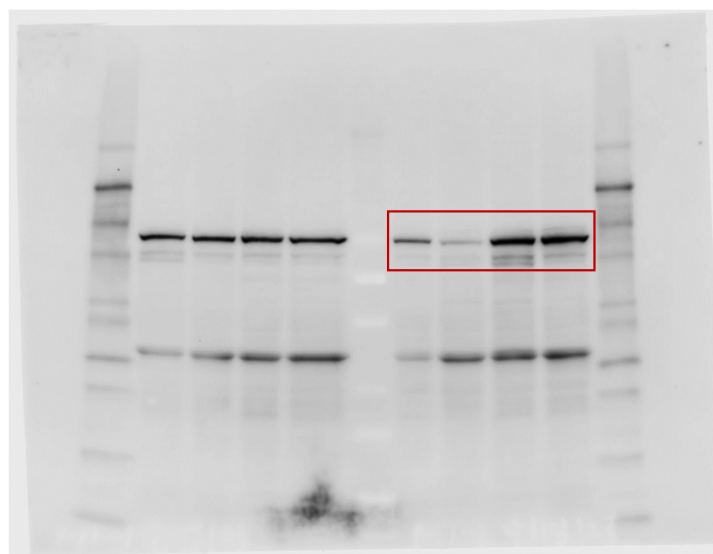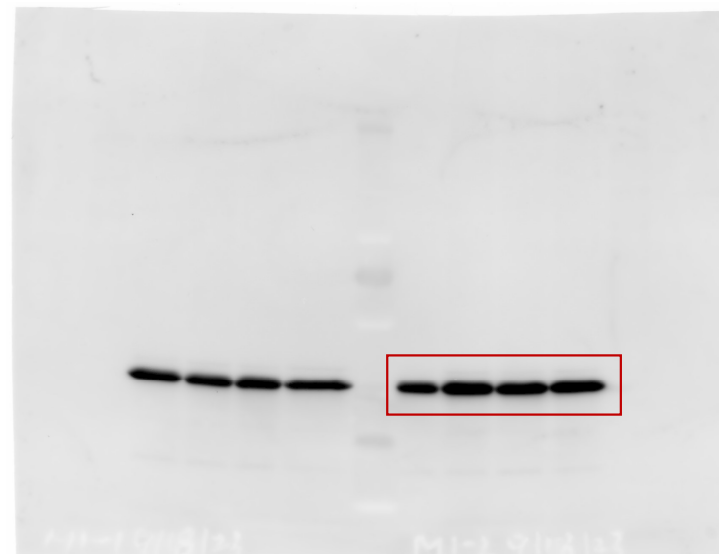

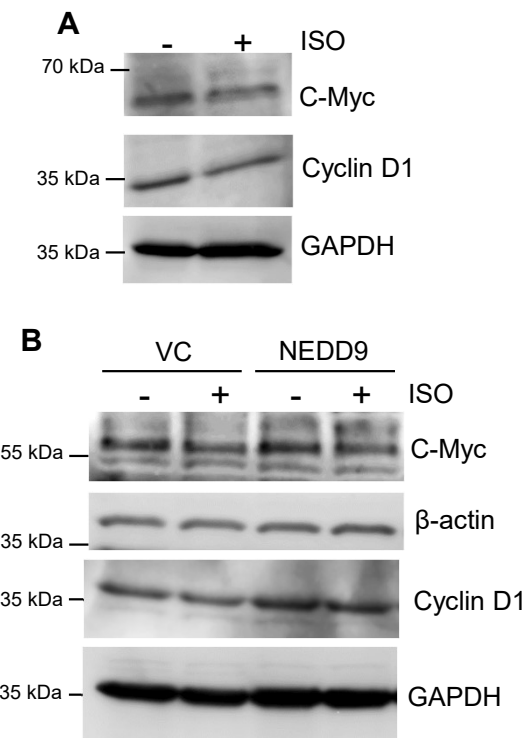

Supplement Figure S1

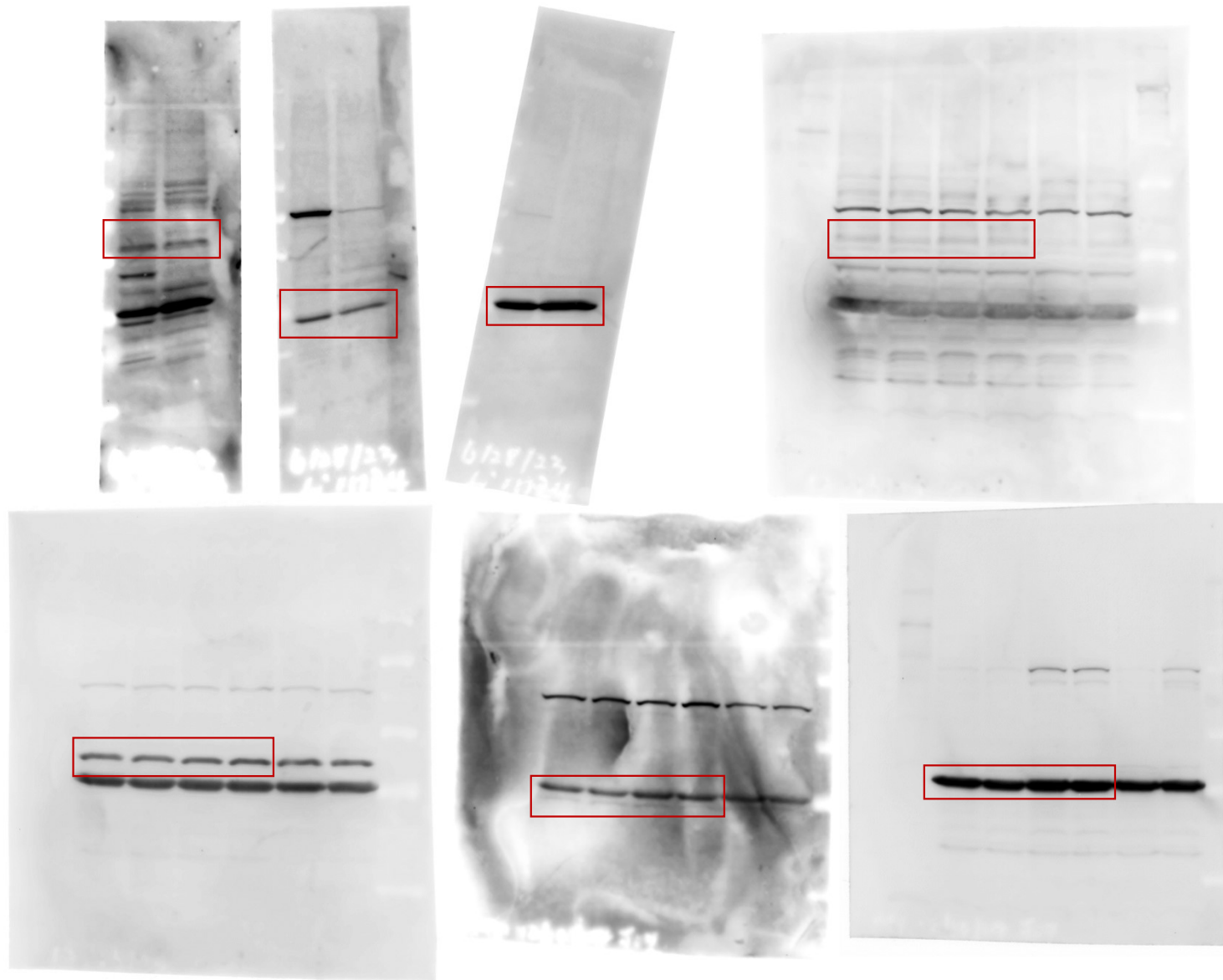

Supplement: Supplementary file 1 [file ijms-26-04207-s001.zip › ijms-3529068-supplementary.pdf]
